# Myxofibrosarcoma-Like Spindle Cell Squamous Cell Carcinoma of the Scalp: A Rare Mimic

**DOI:** 10.7759/cureus.72270

**Published:** 2024-10-24

**Authors:** Eli P Oldham, Svetlana Bobkova, Neil Crowson, Tracy Beswick, Igor Shendrik

**Affiliations:** 1 Office of Medical Student Research, Oklahoma State University Center for Health Sciences, Tulsa, USA; 2 School of Biomedical Sciences, Oklahoma State University Center for Health Sciences, Tulsa, USA; 3 Dermatopathology Section, Pathology Laboratory Associates, Tulsa, USA; 4 Department of Dermatology, Center for Dermatology, Tulsa, USA

**Keywords:** immunohistochemical staining, myxofibrosarcoma, scalp, spindle cell carcinoma, squamous cell carcinoma

## Abstract

We report a challenging case of a man in his mid-70s diagnosed with a myxofibrosarcoma-like spindle cell squamous cell carcinoma (ML-SCC) on the scalp. This rare tumor shares cytologic features with spindle cell squamous cell carcinoma (SCC) with myxoid characteristics but also exhibits architectural elements typical of myxofibrosarcoma, making it nearly indistinguishable by routine light microscopic evaluation. Myxoid spindle cell carcinomas are exceptionally rare, and only one case of ML-SCC has been previously documented in the peer-reviewed medical literature. In our case, the tumor initially presented as an ulcerated lesion with intraepidermal keratinocytic atypia and scattered dermal spindle cells embedded in a myxoid stroma. A deeper biopsy revealed a spindle cell neoplasm with myxoid stroma and curvilinear vessels, features typically seen in myxofibrosarcoma. Immunohistochemical (IHC) staining demonstrated positivity of the spindle cells for p40 and CK5/6, confirming the diagnosis of ML-SCC. This case highlights an unusual morphologic variant of SCC and underscores the importance of IHC evaluation of myxoid spindle cell lesion with markers of epithelial differentiation.

## Introduction

Squamous cell carcinoma (SCC) is the second most common cutaneous neoplasm which can present with variable architectural and morphological patterns, including spindle cell morphology and myxoid features [1]. Myxoid stroma is an infrequent finding in the spindle cell variant of SCC [2], a subtype characterized by spindle-shaped cells with partial or complete loss of typical features of squamous differentiation including intercellular bridges and cytoplasmic keratinization [1]. When spindle cell SCC presents with myxoid features (MSC SCC), it can be difficult to diagnose due to its similarity to spindle cell tumors of mesenchymal derivation and its rarity [3]. A 2023 literature review identified 20 documented cases of MSC SCC [3], with only one case mimicking myxofibrosarcoma (MFS) in the setting of chronic inflammation [4]. Proper evaluation of such tumors using immunohistochemical (IHC) studies is crucial to avoid misdiagnosis and ensure accurate differentiation from soft tissue tumors.

## Case presentation

A man in his mid-70s with an unremarkable medical history presented to the clinic with a lesion on his right mid-medial anterior scalp. He sought medical treatment when the lesion became tender and was uncertain about how long the lesion had been present. Upon clinical examination by the dermatologist, a preliminary diagnosis of SCC was considered, leading to a shave biopsy that measured 0.6 x 0.5 cm.

Microscopic examination revealed an ulcer characterized by a bed of granulation tissue with heavy chronic inflammation. Adjacent to the ulcer epidermis are prominent reactive changes and areas of high-grade atypia, suggesting either a traumatized actinic keratosis or SCC in situ. No definite invasion was identified, but immunohistochemistry with antibodies p40 and CK5/6 demonstrated a few weakly positive cells in the dermis, insufficient for the definite interpretation due to the heavy inflammatory infiltrate obscuring the evaluation. A repeat biopsy was suggested due to the inconclusive nature of the findings.

**Figure 1:**
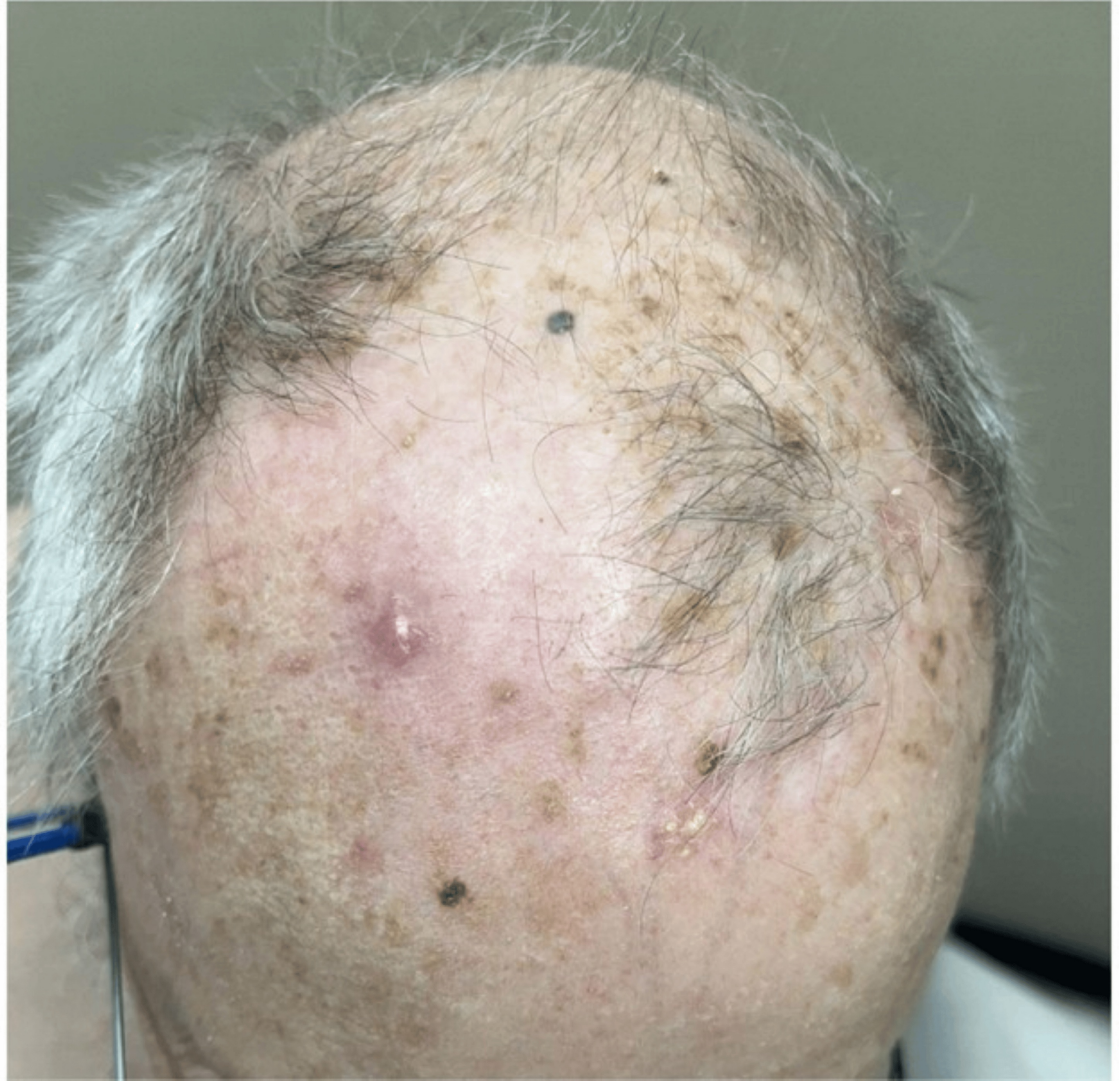
A pink, nodular plaque on the right scalp. The surrounding skin shows extensive photodamage, including actinic keratoses and solar lentigines.

The patient was seen four months after the initial biopsy, presenting with a solitary, well-circumscribed lesion on the scalp (Figure 1). The lesion was a slightly elevated, nodular plaque with a pink to pale coloration. The surface of the lesion appeared smooth with subtle irregularities and without obvious ulceration or crusting. A second biopsy was performed.

**Figure 2:**
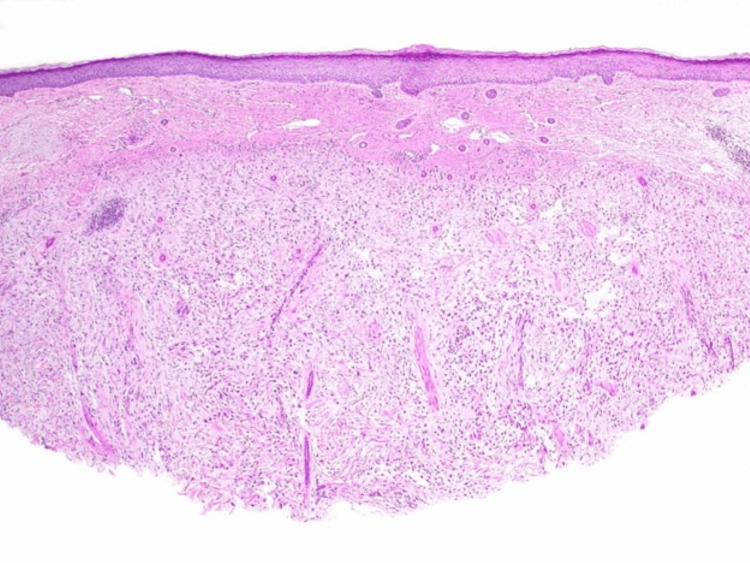
Low-power view showing a spindle cell neoplasm within a myxoid stroma, with a subepidermal scar from a prior biopsy (H&E, x20).

**Figure 3:**
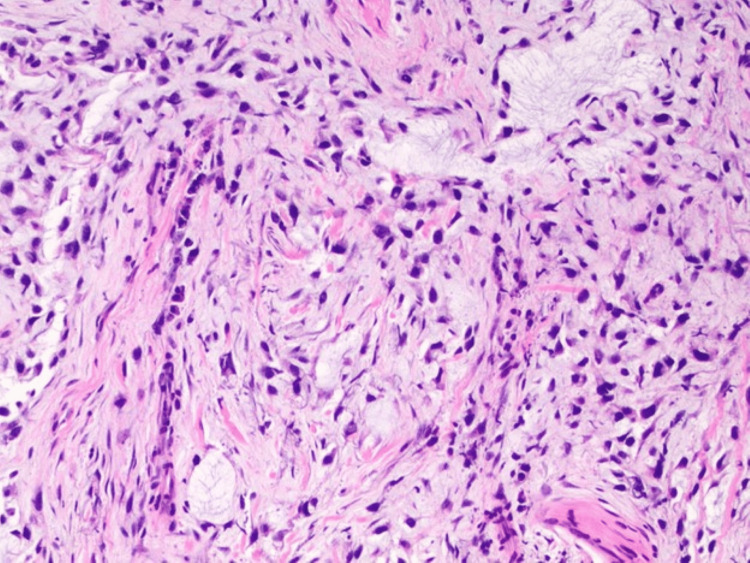
Central region of the tumor displaying curvilinear vessels and stromal mucin pools (H&E, x100).

**Figure 4:**
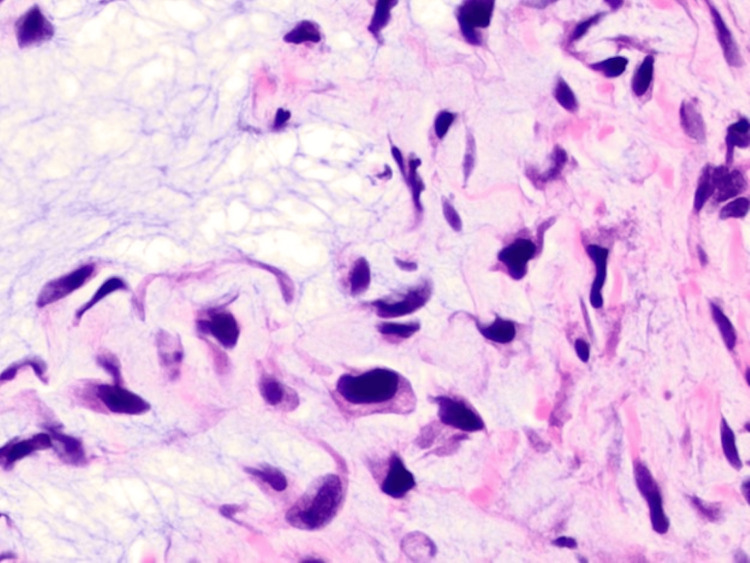
High-power view of a pleomorphic spindle cell neoplasm within a mucinous stroma (visible in the left-hand part of the image) (H&E, x400).

Microscopic examination revealed a dermal-based population of highly atypical spindle cells that were loosely cohesive and sprouted through a thickened collagen table associated with myxoid change and curvilinear vessels (Figure 2 and Figure 3). Mitotic figures, including atypical forms, were prominent (Figure 4). The overlying epidermis was benign and separated from the tumor by fibrosis, consistent with a scar from the previous biopsy (Figure 2).

**Figure 5:**
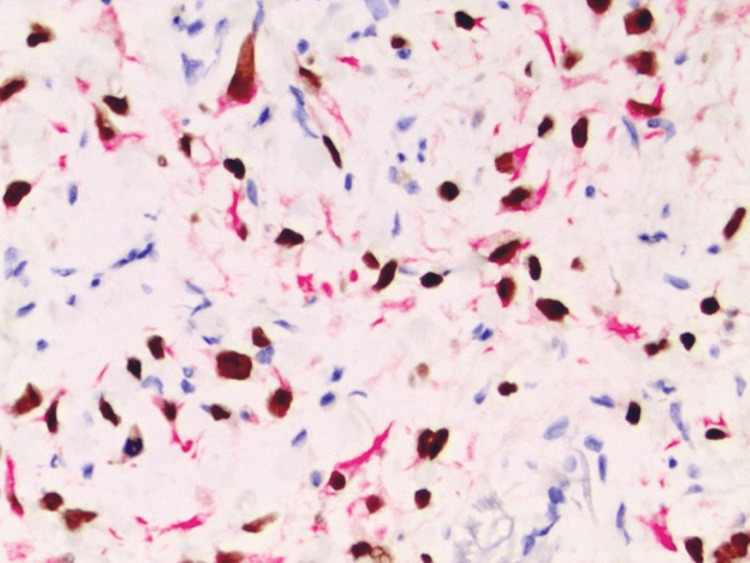
Double immunohistochemical staining showing p40 (dark nuclear stain) and CK5/6 (red cytoplasmic stain) (H&E, x200).

The IHC studies revealed the lesion to be positive for p40 and CK5/6 (Figure 5) while also being weakly positive for CD10. The lesion was negative for AE1/3 cytokeratin, Melan-A, SOX-10, and smooth muscle actin. The pattern of staining was considered diagnostic of spindle cell SCC.

Approximately two months after diagnosis, the lesion was excised to generate a 5.0 x 3.1 x 0.7 cm tissue element with no residual tumor identified by routine light microscopic and IHC examinations. Periodic follow-up visits to assess for local recurrence were recommended. 

## Discussion

MSC SCC was described by Yang et al. as a variant of SCC characterized by poorly differentiated spindle cells and myxoid stromal changes in over 50% of the lesion. The lesion must show expression of at least one cytokeratin marker while being negative for melanocytic and mesenchymal markers [2].

MSC SCC is distinct from other subtypes of mucin-producing cutaneous and mucocutaneous SCCs [2]. Unlike adenosquamous SCC, which contains mucin-producing glandular structures and luminal cells with intracytoplasmic mucin, MSC SCC lacks these features [2]. Adenosquamous carcinoma, a rare and aggressive SCC variant, features squamous cells mixed with mucin-producing cells and sometimes glandular structures [2]. In contrast, MSC SCC is characterized by a mucinous stroma that stains positively with Alcian blue, but true epithelial mucin is absent [2]. In some MSC SCC cases, signet ring-like cells are present but lack intracytoplasmic mucin [2].

As defined, MSC SCC raises histologic differential diagnosis with several sarcomas, including atypical fibroxanthoma, pleomorphic stromal sarcoma, myxofibrosarcoma, and melanoma with myxoid stromal changes. While MSC SCC typically lacks a distinct architectural structure, a variant histologically identical to MFS has also been described [5]. The salient features of MFS include the presence of atypical cells in the myxoid stroma and curvilinear vessels [6], a key feature that is typically absent in other neoplasms including the majority of MSC SCC.

In our report on ML-SCC, the presence of curvilinear vessels made it indistinguishable from MFS on histological evaluation. The IHC markers p40 and CK5/6 were crucial in establishing diagnosis. It should be emphasized that ML-SCC may be positive only with some of the epithelial markers and negative with AE1/3, as it was in our case [2].

The clinical setting of MFS and MSC SCC is somewhat different, with MFS typically presented as a slow-growing, painless mass in the extremities of elderly patients with rare occurrences on the trunk, head and neck, hands, feet, and retroperitoneum [6]. MFS typically arises in patients in their sixth to eighth decades of life and shows a slight male predominance, with cases in patients under 30 being exceptionally rare [6]. Macroscopically, MFS is characterized by multiple gelatinous or firmer nodules with infiltrative margins in deeper lesions [6]. Clinically, it manifests as a slowly enlarging, painless mass with a high propensity for local recurrence [6]. In contrast, MSC SCC often manifests as a nodular or fungating mass on sun-exposed areas, particularly the head, neck, and extremities of elderly individuals [3].

Despite the scarcity of reported cases of ML-SCC, its prevalence may be higher than reported due to the inclusion of this tumor in cases of spindle cell SCC, poorly differentiated SCC, NOS, and, possibly, MFS which escape proper diagnosis due to lack of awareness leading to underutilization of IHC stains. The distinction between MFS and ML-SCC is crucial for proper management, as MFS has a high local recurrence rate but rarely metastasizes [6], whereas ML-SCC may exhibit a more aggressive behavior with metastatic potential [3].

## Conclusions

We describe a rare form of myxoid spindle cell SCC histologically simulating myxofibrosarcoma. The salient clinical, histologic, and IHC features of this tumor are discussed. Heightened awareness of this rare, and likely underdiagnosed, type of SCC is crucial for the evaluation of the tumor, with epithelial markers such as p40 and/or CK5/6 leading to the correct diagnosis.
